# We need to talk about critical care in Brazil

**DOI:** 10.1016/j.clinsp.2022.100096

**Published:** 2022-08-29

**Authors:** Luiz Alberto Cerqueira Batista Filho, Varinder Kaur Randhawa, Alexandre Toledo Maciel, Marcelo Rocha Coimbra

**Affiliations:** a Imed Group, São Paulo, SP, Brazil; b Faculty of Medicine, University of Toronto, Toronto, Canadá

The ongoing COVID-19 pandemic has led to millions of deaths worldwide, and wreaked havoc havoc in all aspects of our lives over the last 2 years. With a population of more than 210 million people, Brazil holds the second largest death toll related to the pandemic, at over 650,000 as of March 2022. Additionally, Brazil accounts for the third-most number of cases worldwide.^1^ While these are impressive numbers, more staggering are the findings of a January 2021 publication reporting that patients with severe COVID-19 pneumonia admitted into Brazilian Intensive Care Units (ICUs) would die at rates as high as 80%.^2^

Many countries have been facing difficulties with the lack of Intensive Care Unit (ICU) beds and specialized health care workers. Despite all the politicization involved with the pandemic, COVID-19 has demonstrated that Low-and-Middle-Income Countries (LMICs) do not possess intensive care settings prepared to deal with critically ill patients, and Brazil is no exception. There is a lack of ICU beds in the majority of Brazilian territories, a problem that has only been highlighted by the pandemic. Even if the patient is lucky to be admitted into an ICU bed, chances are the unit will not have a specialized team, reducing dramatically the chances of survival. Critical care is a health care specialty that has earned little governmental and public attention in Brazil. Even so, it is a fundamental part of bed flow at any hospital, and it will play a bigger role in the years to come. In order to address this problem, it is important to understand how High-Income Countries (HICs) have reduced mortality rates in critical care settings and to analyze how most Brazilian ICUs look after their critical care patients.

## Urban regionalization of critical care beds in Brazil impacts access to care

**Fig. 1:**
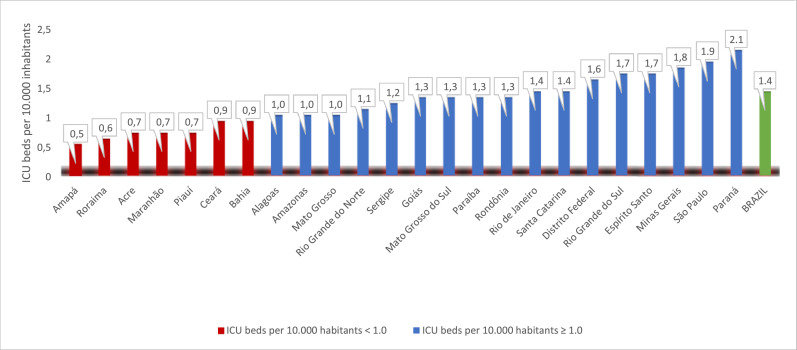
ICU Beds in the Brazilian Public Health System (SUS) per 10.000 inhabitants of each state of the Brazilian Federation. Source: AMIB (2020).

**Fig. 2:**
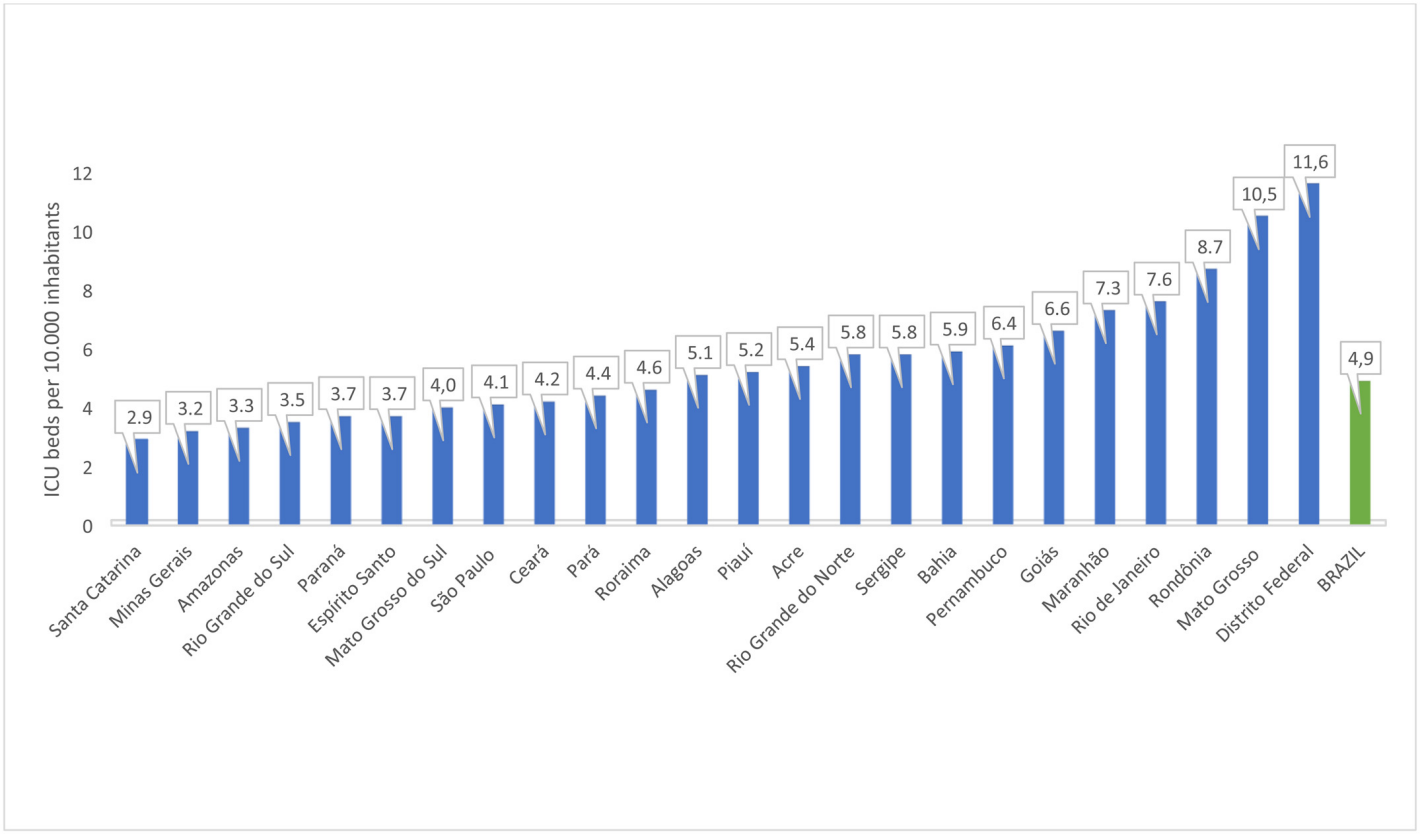
ICU beds in the Private Sector of the Brazilian Health System per 10.000 inhabitants of each state of the Brazilian Federation. Source: AMIB (2020).

**Fig. 3:**
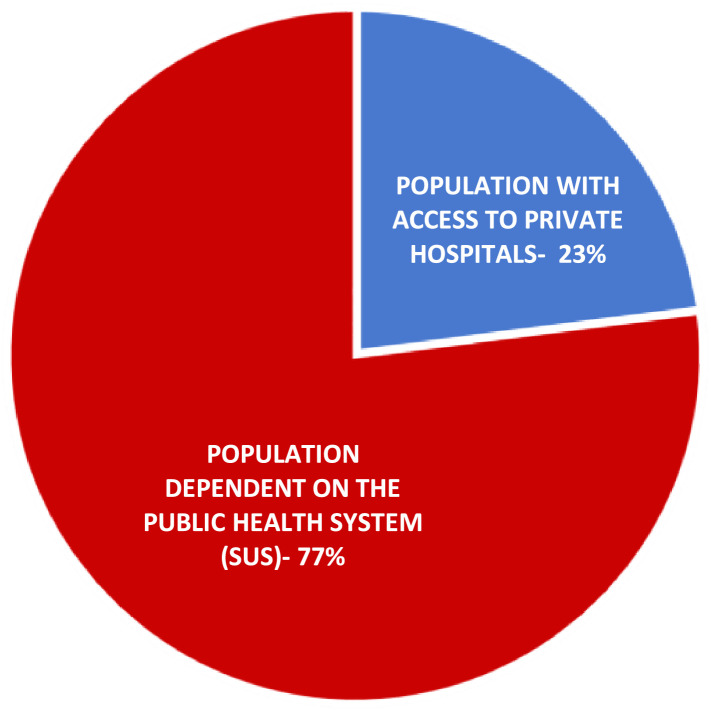
Proportion of the Brazilian population with access to health insurance and private sector ICUs. Source: ANS (2021).

**Fig. 4:**
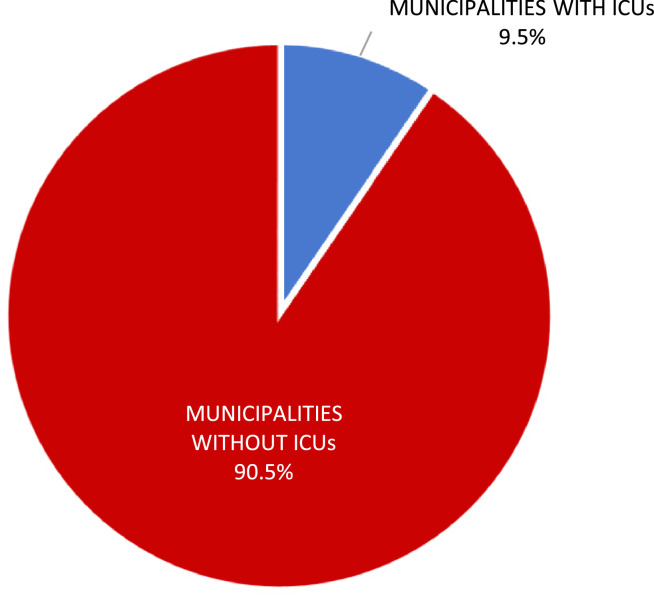
Proportion of municipalities with access to an ICU in Brazil. Source: Conselho Federal de Medicina (2018).

According to the Associação de Medicina Intensiva Brasileira (AMIB), which is the Brazilian Association of Intensive Care Medicine, the Brazilian public health system, called Sistema Único de Saúde (SUS), offers a ratio of 1.4 ICU beds per 10,000 habitants (Fig. 1), against a 4.9 ratio in the private sector (Fig. 2). Combined, both sectors offered a 2.2:10,000 ratio in all Brazilian territory as of 2020. These ratios are considered adequate according to the World Health Organization, which recommends numbers between 1 and 3.^3^ Notably, however, 77% of Brazilians do not have access to health insurance (Fig. 3), and as a consequence, cannot be admitted into a private hospital ICU.^4^ These beds are irregularly distributed since less than 10% of the Brazilian municipalities have ICUs. A study conducted by the Conselho Federal de Medicina (CFM), which is the regulatory body for physicians in Brazil, revealed that in 2018 only 532 of 5570 municipalities in the country have hospitals with critical care beds (Fig. 4), and the number drops to 466 when considering exclusively the public health system. There were ∼43,000 ICU beds in Brazil before the COVID-19 pandemic, with 21,000 (49%) in the public health system and 22,000 (51%) in the private health sector in 2018.^5^ In 2022, these numbers have probably changed, since the pandemic has created 20,000 new ICU beds, but it is hard to say how many effectively remained. The Ministry of Health determined that 6500 of these beds should be converted into regular ICU beds for SUS.^6^

During the pandemic, there were consistent reports about the lack of beds for critically ill COVID-19 patients, especially in smaller cities and poorer states.^7^ Many had to be transferred to big centers in the capitals, either by ambulance or plane, which increases morbidity. Some hospitals created critical care beds on an emergency basis, in order to meet the flood of patients with respiratory failure caused by COVID-19. The capacity of these ad hoc critical care beds may have been variable depending on the resources available at a given center.

## The critical care physician: who takes care of the Brazilian ICUs?

The majority of Brazilian ICUs do not have a critical care specialist physician staffing their units. Unfortunately, such critical care trained professionals are rare, and they tend to be concentrated in big urban areas, and in the richest states of the Brazilian federation. According to AMIB, Brazil currently holds around 6500 critical care specialists, and more than 40,000 would be necessary to manage all the ICU beds in the country.^8^ As expected, the most educated professionals will seek the best hospitals and most advantageous job offers, as it is very difficult to enroll them in peripheric services outside of the capitals despite the salary bid. A good private hospital sometimes has a fully specialized medical staff in their ICU, while some big public hospitals in the peripheral areas of the capital might not even have one critical care specialist, even though these institutions will take care of the sickest patients in the country.

In contrast to ICUs in HICs, in Brazil, it is not necessary to be a critical care specialist to work as a physician in an ICU. At times, the physician that will take care of the public and peripheral ICUs is less experienced (e.g., new medical school graduates) or less specialized (e.g., older physicians who were graduates of specialties unrelated to critical care), and without formal education in critical care medicine. Critical care work is often considered by many as a means towards additional financial remuneration. Without these critical care specialists attending, ICUs rarely have efficient protocols or multidisciplinary rounds, which are the cornerstones of any critical care unit. Recognition of critically ill patients becomes deficient, as many important measures may be taken too late, or never. This may lead to missed opportunities for time-dependent management of critical illness pathologies, such as proning in the setting of acute respiratory distress syndrome, or adequate fluid resuscitation for septic shock. Additionally, there may be a lack of ability to safely perform procedures or to determine when to carry them out since these are not skills often honed by newly graduated doctors or non-specialists. Therefore, the critical care specialist facilitates safe and efficient care with their ability to organize focused multidisciplinary rounds that address the disease pathophysiology and ICU trajectory along with other aspects germane to patient care. This includes mechanical ventilation, enteral feeding, stress ulcer prophylaxis, deep vein thrombosis prophylaxis, early mobilization, and medical reconciliation. The critical care specialist is also well versed in carrying out commonly required procedures in the ICU setting while ensuring the use of critical care bundles.

Unfortunately, as highlighted by the pandemic, the critical care-trained physician is a scarce commodity.^9^ It is not possible in all countries including Brazil to only hire critical care physicians to provide care in the ICU setting. As one solution, some ICUs resorted to employing non-specialists to address this shortage, because they are low-cost, and will not demand big changes in the *status quo*. However, this does not address the issue of having physicians with the required skillset to provide sufficient care within the ICU. As a result, many countries offered larger salaries to the critical care professionals to provide more care during the pandemic. But this decreases the longevity of the critical care physician – this specialty is already considered one of the most stressful in medicine, with the highest rates of burnout – and such crises add to this burden.^10^ Another potential solution to this scarce resource would be to increase the pipeline of graduating medical students with exposure to critical care training. But this is marred by the fact that medical schools provide little time in the ICU and that the specialty is considered underpaid and with excessive workload, and thus less appealing to the newly minted graduate. Often physicians tend to choose this path after having some time practice in another specialty, by developing the desire to deal with the illest patients in the hospital. Paradoxically, many of these students will find their first jobs exactly in the emergency departments of hospitals and at ICUs.

## The nursing team: a major discrepancy between two different worlds

**Fig. 5:**
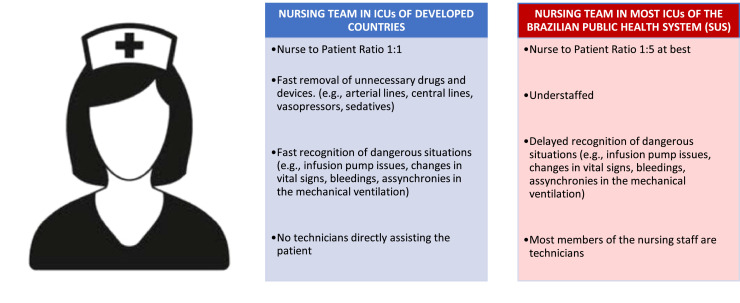
Major differences in the nursing team of developed countries ICUs and Brazilian ICUs.

In Brazil, most ICUs have a nurse-to-patient ratio of 1:5.^11^ All the other members of the nursing team are nurse technicians, with a ratio of 1:2 at best. Nurse technicians are not graduated from universities, as they go through a technician-professionalizing course of 18 months length. They need to be supervised by nurses at all times when taking care of critical patients, and they are not empowered to make decisions in emergency situations. In HICs, such as Canada and the United States, this ratio is 1:1, and there are no technicians. Each patient has their own specialized critical care nurse responsible for coordinating every detail related to care, including modifying prescriptions according to the intensivist's orders. This also allows for swift recognition of changes in a patient's status, and of issues with infusion pumps, lines, or ventilation. This helps to potentially reduce the morbidity and/or mortality that can be associated with prolonged ventilation, vasoactive agent administration, or invasive devices (Fig. 5). While developing countries do not have the same financial resources as developed nations, certainly increasing the vigilance over critical care patients may be one step closer towards mitigating adverse events through early recognition and facilitating the recovery of patients with early weaning and mobilization. If the goal is to reduce mortality and increase the quality of care, managers should consider at least increasing the number of nurse technicians.^12^ Nurses should also be encouraged to do a residency, as a way of improving quality.

## Conclusions

Even though most people could have in mind that the biggest contrast between LMICs and HICs critical care units would be technology, what really makes a difference is human resources. Having a specialized multidisciplinary team well-versed in all aspects of intensive care is crucial for critically ill patients. The COVID-19 pandemic has taught us that simply sending mechanical ventilators to poverty-stricken places like Africa is not enough.^13^ In the same way, solely increasing ICU bed capacity is not sufficient in preventing patients from dying, as evidenced by the extremely high mortality rates reported in the Brazilian critical care units. By contrast, some Brazilian ICUs staffed by critical care-trained physicians have managed to reduce mortality rates to as low as 16.2% for intubated patients with severe COVID-19 pneumonia.^14^

Every crisis presents an opportunity for improvement. Learning from what happened with the pandemic is imperative since critical care will play a fundamental role in healthcare in the years to come. Aside from the fact that the population is increasingly getting older, there are new treatments and drugs being developed that will boost the complexity of patients. The best hospitals already have intensivists not only in the ICU but in all sectors of hospitals. Rapid response teams provide treatment for acutely debilitating patients at any part of the hospital, as the ICU is now considered a unit without a wall.^15^ Critical patients can be found in any sector, anytime, and specialized doctors should be available to take care of them.

Most Brazilian health care institutions are far from this reality, but facing this problem is urgent, as more attention should be given to critical care by hospital managers, governmental institutions, and even medical schools. It is common for students to spend more time in specific specialties, such as ophthalmology and otolaryngology, than learning about critical patients, which is more useful for the vast majority of them. Increasing the number of ICU beds in SUS is also necessary so that the overcrowded emergency departments at the public hospital could have relief, and patients have better care. After the pandemic, it is clear the value of a good ICU bed. Unfortunately, new pandemics will be on the way,^16^ and it is better to be prepared.

## Declaration of Competing Interest

The authors declare no conflicts of interest.
